# An insight into polyscopoletin electrosynthesis by a quality-by-design approach

**DOI:** 10.1007/s10853-022-07349-8

**Published:** 2022-06-22

**Authors:** Riccardo Goldoni, Douglas Vieira Thomaz, Tiziano Di Giulio, Cosimino Malitesta, Elisabetta Mazzotta

**Affiliations:** 1 Department of Electronics, Information and Bioengineering (DEIB), Politecnico Di Milano, 20133 Milan, Italy; 2 National Research Council, Institute of Electronics, Computer and Telecommunication Engineering (CNR-IEIIT), 20133 Milan, Italy; 3 Laboratorio di Chimica Analitica, Dipartimento di Scienze e Tecnologie Biologiche e Ambientali (Di.S.Te.B.A.), Università del Salento, 73100 Lecce, Italy

## Abstract

Scopoletin (SP) as a functional monomer for electropolymerization has recently been investigated in the context of molecularly imprinted polymers for biosensing applications. Herein we describe an in-depth analysis of the mechanisms involved in the electropolymerization of SP toward the optimization of the experimental conditions for applications in sensor studies. PolySP films have been in situ synthesized on a standard glassy carbon electrodes by varying three independent experimental parameters, and the output of the analysis has been evaluated in terms of the resulting electroactive area and surface coverage. A quality-by-design approach including design-of-experiments principles and response surface methodology produced unbiased observations on the most relevant parameters to be controlled during the electropolymerization of SP. By combining the output of electroactive area and surface overage, we highlighted a strong dependence on the monomer concentration and scan rate. Thus, an appropriate selection of these two parameters should be sought to have an optimal electropolymerization process, leading to uniform films and homogeneous surface behavior. This study shows that the application of multi-factorial analysis in a comprehensive design of experiments allows the systematic study of polymer electrosynthesis. Therefore, this research is expected to guide further efforts in the electropolymerization of several functional monomers.

**Supplementary Information:**

The online version contains supplementary material available at 10.1007/s10853-022-07349-8.

## Introduction

**Figure 1:**
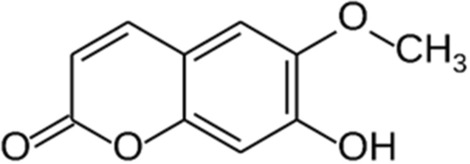
Structural formula of SP

Scopoletin (SP), also known as 7-hydroxy-6-methoxy coumarin, is a natural compound, with a wide spectrum of biological functions [1], isolated for the first time by Eykman in 1884 [2]. It belongs to the group of coumarins that are derivatives of benzo-α-pyrones [3] (Fig. 1).

SP can be found in nature, mainly in plant species, where its levels change in response to certain stresses such as exposure to heavy metals, irradiation with UV light [4], or as a result of the occurrence of some phytopathologies [5, 6] Although it is not clear what metabolic pathway leads to the increase in SP concentrations, that seems to be related to self-protection mechanisms and pathogen resistance. SP is, in fact, recognized as a phytoalexin [1, 4] with antimicrobial action.

SP shows various pharmacological properties [1, 7], such as anti-inflammatory and antioxidant activity as well as scavenging of reactive oxygen species. Moreover, it has been reported to have beneficial effects in the case of neurodegenerative disorders, including Alzheimer's disease and Parkinson's disease [7–9]. Due to its great potential, an increasing number of research activities have been focused on better investigating the properties of SP. As an example, in a recent work it is hypothesized that the intake of SP after the occurrence of autoimmune diseases such as multiple sclerosis significantly improves the severity of the disease and prominently decreases inflammation and demyelination of central nervous system [8]. Although these tests were conducted on animals, further developments may indicate their potential use in the treatment of similar diseases in humans.

The use of SP as a raw material for the development of electrochemical sensors started with the seminal work of Gajovic-Eichelman et al. [10] who, about 20 years ago, discovered that this simple compound can undergo a process of electropolymerization, which results in the formation of insulating polymeric films. Since then, SP has been used as a functional monomer for the electrosynthesis of polymeric films, which are mainly employed in the field of molecularly imprinted polymers (MIPs). Briefly, MIPs are synthetic materials that have selective sites within them for molecular recognition of targets (or templates). These “plastic antibodies” are produced by polymerizing functional monomers in the presence of the target, acting as the template. Subsequently, the template is removed with formation of imprinted cavities, complementary to the target and thus endowed with molecular memory [11–17]. Pioneering work in the use of SP for the synthesis of MIPs were those of the group of Prof. Scheller, who successfully electrosynthesized SP-based MIP on different supports [18–20]. Subsequently, several proteins have been used as templates for the development of MIP-based sensors, such as laccase [20], cytochrome C [21], human serum albumin and ferritin [22], transferrin [19], lysozyme [23] and even SARS-CoV-2 spike protein [24].

The popularity of SP [25, 26] as a monomer for the synthesis of sensitive polymers is due to its easy polymerization [10], low cost and solubility in water [27], allowing the use of aqueous solutions instead of toxic and hazardous solvents [23]. Such polymers are still to be considered niche materials since their application outside the field of MIPs has not yet been explored, and therefore, its potential has not been fully expressed. However, the work by Di Giulio et al. [23] laid the foundation for the utilization of polySP-based MIP sensor in clinical applications. This includes salivary biosensors, which are receiving great attention as potential candidates for non-invasive diagnostics [28–30].

Two main approaches are used for SP electropolymerization, according to the literature: cyclic voltammetry (CV) and amperometry technique (AT). Some of the most representative examples are shown in Table 1.

Often, as observed in Table 1, the experimental parameters vary from one work to another, without a rational explanation from the authors as to why they were specifically selected. Here, we propose the use of the quality-by-design approach [34], for the rational optimization of the experimental conditions of electrochemical synthesis of SP, based on the critical evaluation of the response variables (electroactive area and surface coverage) obtained after the execution of the planned experimental design. Electroactive area was herein selected as a dependent variable because of its effects on electric current and the implication of this parameter in sensing applications. Moreover, it can be easily calculated using the response of a redox probe such as ferrocyanide and serves as a reliable descriptor of the formation of insulating coatings atop electrode surfaces. Similarly, surface coverage was also selected as a dependent variable due to its descriptive potential in regard to the stacking of polymer films on conductive materials and also to provide an additional descriptor of changes on electrode surface which could be compared to the electroactive area. To test this approach, we chose to investigate the electrosynthesis of SP by cyclic voltammetry, which among the two most explored electrochemical techniques for the synthesis of this polymer is the one for which the experimental parameters are more susceptible to change: namely, monomer concentration, scan rate and the number of cycles. Nonetheless, the systematic study of the influence of these parameters on (electro)polymerization reactions is seldomly explored in the literature [35]. Therefore, this work is, to the best of our knowledge, the first report detailing a comprehensive multifactorial study of polyscopoletin electrosynthesis employing thoughtful design of experiments, multivariate analysis and response surface methodology.

**Table 1:** Representative examples of polySP electrochemical synthesis

Electrochemical technique	Experimental conditions	Monomer concentration	Working solution	References
CV	Applied potential: 0.2–0.7 V; scan-rate: 50 mVs^−1^; no. of cycles: 3	2 mM	50 mM NaCl solution in water	[10]
CV	Applied potential: 0–1 V; scan-rate: 150 mVs^−1^; no. of cycles: 5–20	0.5 mM	100 mM NaCl solution in water	[20]
CV	Applied potential: 0–1 V; scan-rate: 150 mVs^−1^; no. of cycles: 3	1 mM	Phosphate buffer solution (PBS)	[22]
CV	Applied potential: 0–1 V; scan-rate: 20 mVs^−1^; no. of cycles: 3	1 mM	100 mM NaCl solution in water	[21]
AT	Applied pulsed potential: 0 V for 15 s; 0.5 V for 35 s vs Ag/AgCl; Pulse pairs: 30	0.5 mM	Phosphate buffer solution (PBS)	[31]
AT	Applied pulsed potential: 0 V for 5 s; 0.7 V for 1 s; Pulse pairs: 30	0.5 mM	10 mM NaCl and 5% v/v ethanol in water	[32]
AT	Applied pulsed potential: 0 V for 5 s; 0.9 V for 1 s; Pulse pairs: 30	0.5 mM	10 mM NaCl solution in water	[33]
AT	Applied pulsed potential: 0 V for 5 s; 0.9 V for 1 s; Pulse pairs: 50	0.5 mM	100 mM NaCl solution in water	[18]

## Materials and methods

### Reagents and solutions

The reagents used in the study were analytical-grade chemicals purchased from Sigma-Aldrich. All solutions were prepared in ultra-pure water. The chemical reagents used included potassium hexacyanoferrate (III), K_3_[Fe(CN)_6_], potassium hexacyanoferrate (II) K_4_[Fe(CN)_6_] and SP 95%. Phosphate buffer saline (PBS) solutions (50 mM, pH 7.4) were prepared by dissolution of the commercial MSP and DSP in appropriate proportions, adding NaOH 5 M to adjust the final pH. The [Fe(CN)_6_]^3−/4−^ solution was prepared in 5 mM PBS containing 0.1 M KCl. The SP solutions were prepared in PBS at a concentration of 1, 1.5 and 2 mM.

### Electrochemical apparatus

A standard electrochemical cell comprising three electrodes and a PVC cap was used. A glassy carbon electrode (GCE) was used as working electrode, a saturated calomel electrode (SCE) was used as reference electrode, and a platinum wire was used as auxiliary electrode. All electrodes were purchased from CH Instruments (Tennison Hill Drive, AU, USA). Electrodeposition and electrochemical characterization were performed with a portable potentiostat/galvanostat, PalmSens, EmStat4 Blue. The PSTrace 5.8 software (PalmSens, Houten, Netherlands) was used to run the experiments and automatically gather and store the experimental data.

### Electrodeposition of polySP on GCE

Electrodeposition of polySP films was obtained through cyclic voltammetry (CV) in solutions containing different concentrations of SP monomer. Initially, the working electrode is cleaned through mechanical polishing on pads containing a solution of alumina slurry of size 0.3 µm. The electrode is then rinsed with deionized water. CVs are performed in a potential window from 0 V to + 1 V vs. SCE, scanning toward positive potentials. The electrode is then rinsed with water before the electrochemical characterization.

### Electrochemical characterization

Electrochemical characterization was performed after each experimental run. The electrical response of the modified surface was investigated with cyclic voltammetry (CV) in a solution of 5 mM [Fe(CN)_6_]^3−/4−^ prepared in 5 mM phosphate-buffered saline (PBS) solution (pH 7.4), containing 0.1 M KCl. The potential in CV measurements was scanned between 0 V and + 1 V at increasing scan rates up to 250 mVs^−1^.

The electroactive area has been calculated using the Randles–Sevcik treatment that correlates the peak currents recorded through a cyclic voltammetry scan with several factors including scan rate, concentration of the electroactive probe (i.e., ferrocyanide) and, indeed, electroactive area.

The Randles–Sevcik treatment is described by Eq. 9, for diffusion-controlled processes:

1 $$i = \left( {2.6865 \times 10^{5} } \right)A D^{1/2} c v^{1/2}$$

where *i*, *A*, *D*, *c* and *v* are the anodic peak current, the electroactive area, the diffusion constant, the concentration of electroactive probe in solution and the scan rate, respectively.

Moreover, Cottrell equation was used to calculate the surface coverage from chronocoulometric data (Eq. 10). This equation describes the time evolution of charge in redox systems as shown below:

2 $$Q = \frac{{2nFAD^{\frac{1}{2}} C_{0} t^{\frac{1}{2}} }}{{\pi^{\frac{1}{2}} }}$$

where *n*, *F*, *A*, *D*, *C*_0_ and t are the number of electrons transferred, the Faraday’s constant, the electrode area, the diffusion coefficient and the concentration of the analyte (i.e., the monomer) in solution, respectively.

The surface coverage (Γ) can be drawn from Cottrell equation according to the following relationship (Eq. 11):

3 $$\Gamma = \frac{Q}{nFA}$$

While similar to the Randles–Sevcik equation, the integrated charges discriminated by Cottrell equation allows the correlation of the surface coverage with the concentration of the functional monomer in solution, which is particularly useful when studying electropolymerization processes.

### Design of experiments

Design of experiments (DoE) is a systematic and rigorous approach that is used to study a system and determine the correlation among the factors of the system and the effects of each factor. The quantification of the error that causes the variability in the system is based on residual sum of squares. The DoE was used to optimize and evaluate the main effects of each factor, as well as their interactions and quadratic effects.

In this work, a Box–Behnken experimental design (BBD) based on a 3^3^ matrix was used [36]; thus, three factors were selected and three levels were tested for each factor. The Box–Behnken experimental designed was selected, among others, due to the high reproducibility of its model, despite having limited capacity of predicting borderline nonlinear design spaces [34, 36]. Notwithstanding, the Box–Behnken experimental design often showcases better fitting than other multifactorial approaches, such as the 3^3^ full factorial, central composite and Doehlert design [36].

The factors herein studied were scan rate (*A*), number of cycles (*B*) and concentration of the monomer (*C*), and they were all set as continuous independent variables. These factors were selected as they are usually critical key parameters in the experimental design of electro-analytical chemistry protocols. More refined versions of this experimental design could further include solvent type and pH of the solution that we deemed to be parameters of lower value for a preliminary study.

For each factor, three equidistant levels were proposed, based on a literature review of most commonly reported values [10, 18, 20–22, 31–33]. The factors, levels and their respective units are presented in Table 2.

**Table 2:** Factors and levels of the DoE

Levels	Factors		
	*A* (mV/s)	*B*	*C* (mM)
Low level (− 1)	50	5	1
Medium level (0)	100	15	1.5
High level (+ 1)	150	25	2

The number of experiments (*N*) required for the development of BBD is defined as *N* = 2 *k*(*k* − 1) + *C*_0_, (where *k* is number of factors and *C*_0_ is the central point). Accordingly, the Box–Behnken DoE consisted in performing 17 experiments, five of which were replicates of experiments performed at the medium level (*A* = 100, *B* = 15 and *C* = 1.5), namely the center points. The experiments were randomized to minimize bias originated from external factors. Two dependent variables were assessed, namely the electroactive area (EA), herein calculated from the Randles–Sevcik relation, and surface coverage (SC), herein obtained by the integrated charge of the chronocoulometric plots according to the Cottrell relation.

### Analysis of variance

Considering the regression model, described by Eq. 1:

4 $$y_{i} = a + bx_{i} + e_{i}$$

a fitting model is built based on Eq. 2:

5 $$\hat{y}_{i} = \hat{a} + \hat{b}x_{i}$$

where the error is represented by the term $e_{i}$.

The coefficients $a$ and $b$ in Eq. 1 are the coefficients of the regression model while $\hat{a}$ and $\hat{b}$ in Eq. 2 are the coefficients of the fitting model. The divergence between observations and fitting model is represented by the residual sum of squares (SS) that is defined based on Eq. 3:

6 $${\text{SS}} = \mathop \sum \limits_{i = 1}^{X} \left( {y_{i} - \hat{y}_{i} } \right)^{2}$$

Two distinct components make up the value of SS, namely the Lack of Fit and the Pure Error. The Lack of Fit term refers to how closely the model describe the trend in the data points. The second component of the error, the Pure Error, arises due to random variation in the data. If the Lack of Fit is predominant over the Pure Error; then, most probably the model should be modified to better represent the distribution of data points.

The Mean Square of the Error (${\text{MS}}_{{\text{E}}}$) is calculated as follows (Eq. 4):

7 $${\text{MS}}_{{\text{E}}} = \frac{{{\text{SS}}_{{\text{E}}} }}{{df\left( {{\text{SS}}_{{\text{E}}} } \right)}}$$

where $df({\text{SS}}_{{\text{E}}} )$ represent the degrees of freedom of the error.

The Mean Square of the Term (${\text{MS}}_{{{\text{term}}}}$) is calculated as in Eq. 5:

8 $${\text{MS}}_{{{\text{term}}}} = \frac{{{\text{SS}}_{{{\text{term}}}} }}{{df\left( {{\text{SS}}_{{{\text{term}}}} } \right) }}$$

where $df({\text{SS}}_{{{\text{term}}}} )$ represents the degrees of freedom of the term.

ANOVA as a statistical method produces two measures of statistical significance, the *F*-statistic and the *p*-value. These values are inversely correlated with each other.

The *F*-statistic can be computed for each term as (Eq. 6):

9 $$F = \frac{{{\text{MS}}_{{{\text{term}}}} }}{{{\text{MS}}_{E} }}$$

The *p*-value is a threshold value that is used to evaluate the statistical significance of experimental findings. Usually a standard *p*-value < 0.05 is used to determine statistical significance of a specific factor in the analysis [37].

### Response surface methodology

The output of the experiments was used to calculate a response-surface model through second-order polynomial regression, which conveys each dependent variable as a function of the independent variables (i.e., factors in the DoE). The mathematical operation used for the model fitting is described in Eq. 7, while the fitted operation according to the coded variables described in Table 2 is presented in Eq. 8 [36].

10 $$f\left( x \right) = y = \beta_{0} + \mathop \sum \limits_{i = 1}^{k} \beta_{i} x_{i} + \mathop \sum \limits_{i = 1}^{k} \beta_{ii} x_{i}^{2} + \sum \sum \beta_{ij} x_{i} x_{j} + \varepsilon$$

11 $$(f\left( x \right) = y = \beta_{0} + \beta_{i} A + \beta_{i} B + \beta_{i} C + \beta_{ii} A^{2} + \beta_{ii} B^{2} + \beta_{ii} C^{2} + \beta_{ij} AB + \beta_{ij} AC + \beta_{ij} BC$$

wherein y stands for the predicted response, *β*_0_ is the constant coefficient, *β*_i_ is the linear coefficient of the *i*th iteration of the input factor *x*_*j*_, *β*_*ii*_ is the quadratic coefficient of the input factor *x*_*i*_, and *β*_*ij*_ stands for the interaction coefficient between *x*_*i*_ and *x*_*j*_. Furthermore, ε is the error of the model.

## Results and discussion

### Experimental

Experiments were performed following an unrandomized experimental matrix, as reported in the described DoE. The experimental matrix is presented in Table 3, along with the encoded results for EA and SC.

**Table 3:** Experimental matrix of the DoE in standard order

Run #	*A*	*B*	*C*	EA	SC
1	− 1	− 1	0	0.234234	− 0.01959
2	1	− 1	0	0.351351	− 1
3	− 1	1	0	0.704955	1
4	1	1	0	0.753378	− 0.77738
5	− 1	0	− 1	0.925676	− 0.03096
6	1	0	− 1	0.926802	− 0.96819
7	− 1	0	1	− 1	0.612725
8	1	0	1	− 0.55743	− 0.93136
9	0	− 1	− 1	0.968468	− 0.72816
10	0	1	− 1	1	− 0.35822
11	0	− 1	1	− 0.5473	− 0.96222
12	0	1	1	0.079955	− 0.65025
13	0	0	0	0.734234	− 0.54377
14	0	0	0	0.759009	− 0.54561
15	0	0	0	0.88964	− 0.5764
16	0	0	0	0.865991	− 0.55513
17	0	0	0	0.882883	− 0.55393

**Figure 2:**
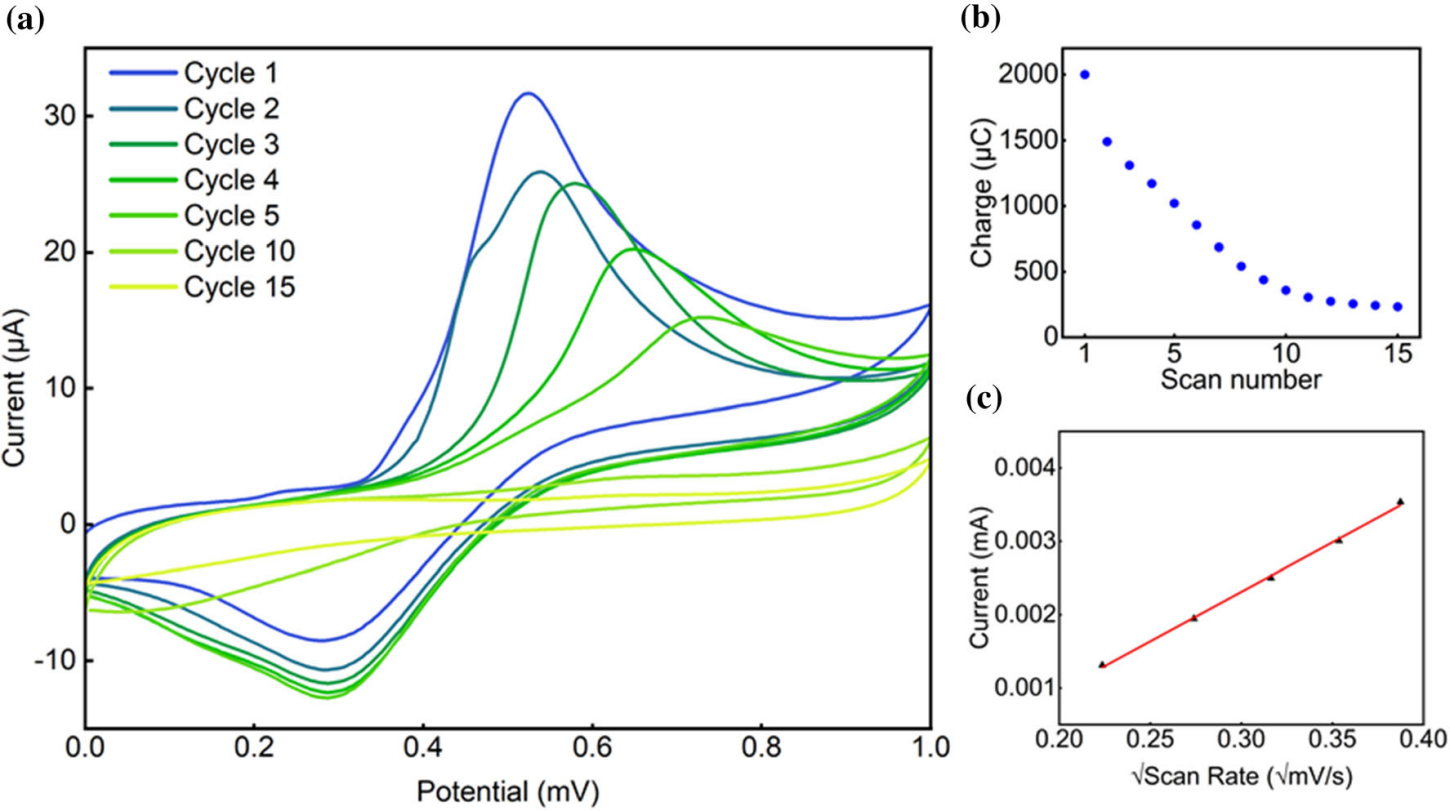
**a** CV curves for SP electropolymerization process in intermediate conditions (*A* = 100, *B* = 15 and *C* = 1.5). **b** Charge vs. scan number graph derived from chronocoulometric plots during electropolymerization, which was used to estimate the surface coverage. **c** Correlation curve between current and square root of the scan rate obtained in ferrocyanide solution on polyscopoletin modified electrode, which was used to estimate the electroactive area. Each experiment yielded two datasets akin to **b** and **c**, which were used to compute the dependent variables herein investigated

SP-based polymers have been electrochemically synthesized on the surface of a GCE by cyclic voltammetry technique, scanning the potential of the electrodes between 0 and 1.0 V, varying scan rate and number of cycles as electrochemical parameters. Such potential program allows monomer oxidation leading to polymerization according to a reaction mechanism that has already been largely documented in literature [10, 31]. As shown in Fig. 2a, the current decreases during the electropolymerization procedure when increasing of the number of potential scans. The highest current intensity was obtained in the first cycle of the polymerization process. Contextually, the anodic peak potential (at ~ 0.52 V for the first scan) shifts to more positive values as the number of cycles increases, and this is presumably related to changes in the electrode surface features. The occurrence of further oxidation processes becomes more difficult upon the formation of an insulating film, therefore leading to shifts toward higher potentials. After 10 cycles, the currents are rather low, and oxidation peaks can hardly be identified. Proceeding further, the voltametric curves do not change dramatically. The magnitude of the current values recorded in these conditions presumably signals the formation of an insulating film, which prevents the charge transfer at the electrode surface (Fig. 2b), in agreement with what has already been largely observed in previous works [23].

### Model validation

Analysis of variance (ANOVA) has been used to validate our model. A three-way ANOVA was used in this framework since the experimental design is based on three factors (*A*, *B*, *C*), each having three distinct levels. Electroactive area (EA) and surface coverage (SC) were chosen as dependent variables to be used in the evaluation of the overall electropolymerization process. A separate three-way ANOVA has been computed for each dependent variable.

Table 4 reports the ANOVA table for the factors that have been investigated for their influence on the resulting electroactive area.

**Table 4:** ANOVA table for factors (EA)

Factors	SS	*df*	MS	*F*	*p*-value
*A* (*L* + *Q*)	0.446981	2	0.223490	41.0204	0.002161
*B* (*L* + *Q*)	0.293401	2	0.146700	26.9260	0.004781
*C* (*L* + *Q*)	5.102142	2	2.551071	468.2351	0.000018
Lack of Fit	0.272620	6	0.045437	8.3397	0.029721
Pure Error	0.021793	4	0.005448		
Total SS	6.211101	16			

*SS* sum of squares, *df* degrees of freedom, *MS* mean sum of squares, *F* variation between sample means, *L* linear component of the factor, *Q* quadratic component of the factor

Table S1 shows the same ANOVA analysis, where linear and quadratic components of each factor have been decoupled. In this case, each factor has been analyzed both in its linear and quadratic component and the respective *p*-value has been computed to provide their individual statistical relevance.

A comparison of the results from Table 4 and Table S1 highlights how factor decomposition allows for a better discrimination of statistically significant effects. Factor *B* (number of scans) as a whole can be considered statistically significant, with a *p*-value of 0.004781. However, after decomposing the factor into its linear and quadratic contribution, only the linear component *B*(*L*) appears to be statistically significant. On the other hand, for the factor *A*, only the quadratic component was significant, while for factor *C*, both linear and quadratic contributed to the model.

Table 5 and Table S2 report the ANOVA table for the factors that have been evaluated with respect to the resulting surface coverage. As described previously, factor decomposition allows to account only for specific components of the factor that has statistical significance. In this case, as it was the case of the electroactive area, factor *B* contributes to the surface coverage only with a linear component *B*(*L*), with the quadratic component *B*(*Q*) having no statistical relevance. We can thus preliminary infer from these first observations that the number of scans affects both outputs of our DoE in a linear fashion. Nevertheless, for the factor *A*, both linear and quadratic components contributed to the model, while for factor *C*, only the quadratic component showcased statistical significance.

**Table 5:** ANOVA table for factors (SC)

Factors	SS	*df*	MS	*F*	*p*-value
*A* (*L* + *Q*)	3.948284	2	1.974142	11720.82	0.000000
*B* (*L* + *Q*)	0.462895	2	0.231447	1374.14	0.000002
*C* (*L* + *Q*)	0.068745	2	0.034372	204.07	0.000094
Lack of Fit	0.482476	6	0.080413	477.42	0.000012
Pure Error	0.000674	4	0.000168		
Total SS	4.946444	16			

The physical meaning of the linear and quadratic contributions to the model can be traced back to the Randles–Sevcik and Cottrell equation. Since each parameter, with the exception of factor *B*, is tied to an element in these equations, it could be suggested that their calculated contribution in the statistical model would mimic that of their mathematical counterparts. In this sense, factor *A* (i.e., scan rate) is present in Randles–Sevcik equation as a quadratic component in regard to the peak current. This justifies the stronger contribution of this parameter to the model, as it follows an expected behavior that is extensively reported in literature. On the contrary, the factor *C*, i.e., concentration of the functional monomer, is linearly correlated with the charge as stated by Cottrell equation. Nevertheless, we have observed quadratic contributions to be statistically significant for the concentration of scopoletin in the electrochemical cell. In this sense, it could be suggested that the role of concentration in the formation of polyscopoletin is not only limited to the predicted linear contributors.

The observed values for each dependent variable are correlated with those predicted by the model, as a procedure to validate the DoE. The comparison between these values allows to draw a correlation line and produce two metrics of fitting that are the R^2^ and the adjusted R^2^, for each variable. The R^2^ is normally used to determine the ability of the model in describing the variation in the experimental data.

**Figure 3:**
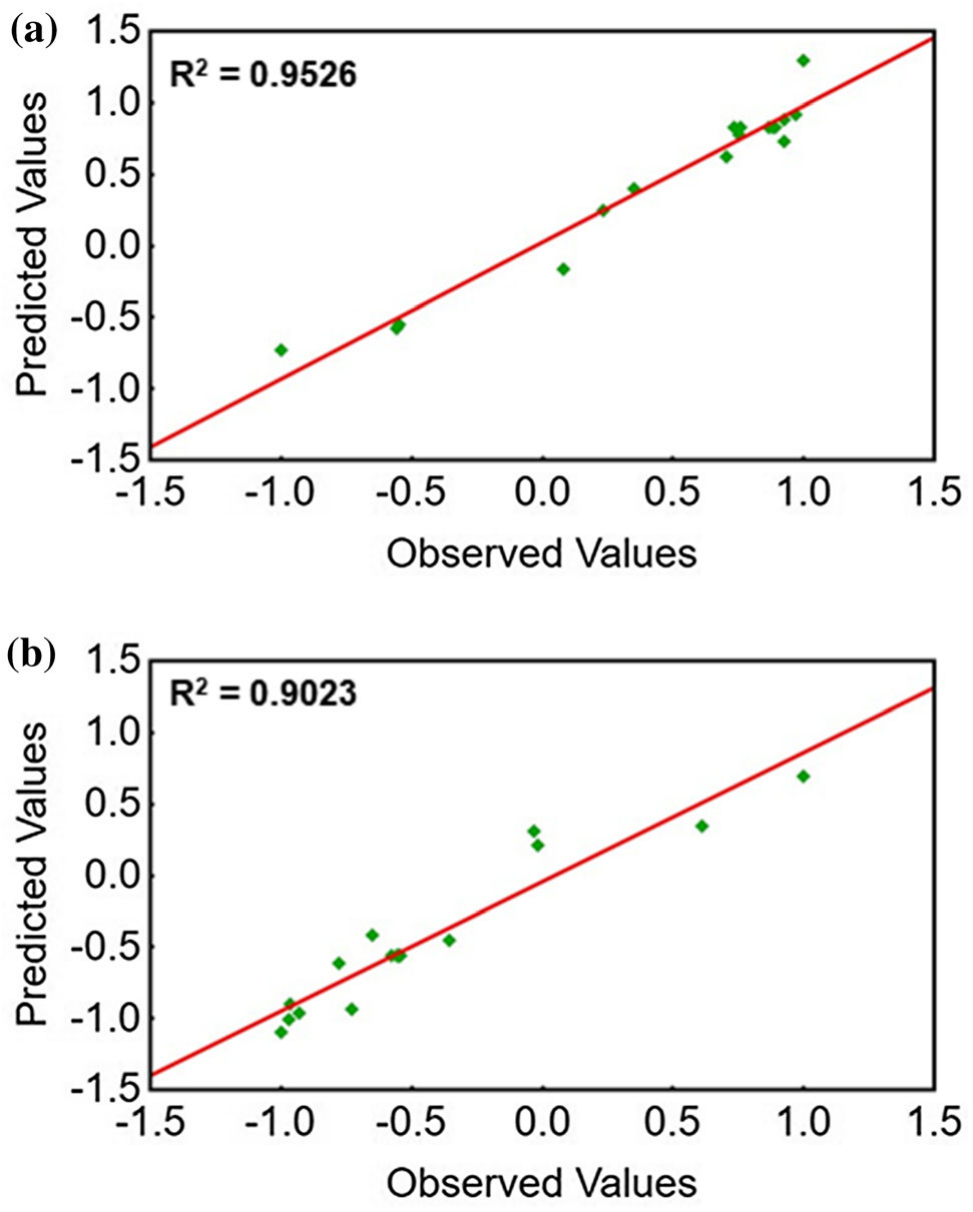
**a** Observed vs. predicted values of the electroactive area; **b** observed vs predicted values of the surface coverage

The plot in Fig. 3a represents the observed values of electroactive area and those predicted by the fitting model. Since the values of the dependent variable have been also encoded in the range -1:1, they are adimensional. The choice to encode the output values in the same fashion as it has been done with the DoE factors allows for a more homogeneous representation of results. Absolute values of the electroactive area and surface coverage are obtained from Table 2 by simply decoding them. The model showcases a remarkable fitting for most of the experimental data points. An *R*^2^ = 0.9526 has been computed for the first dependent variable, with an adjusted *R*^2^ = 0.9242. Adjusted *R*^2^ values can provide a more precise view of that correlation by also considering how many independent variables are added to a particular model.

Figure 3b reports the observed values of surface coverage against those produced by the fitting model. The fitting proves to be reliable with an *R*^2^ = 0.9023 and an adjusted *R*^2^ = 0.8437. These values are lower those obtained in the evaluation of the first variable, thus signaling that the model has a better fitting when considering the electroactive area as dependent output.

These graphs produce preliminary hints into the suitability of the ranges that have been chosen for each factor. This allows our analysis to cover both extremes of the dataset, henceforth leading to a comprehensive model which underwent thorough validation. In Fig. 3a, most of the datapoints are clustered in the top right area of the graph, thereby showing that higher values of electroactive surface area strongly contribute to the predictive power of the model. On the other hand, Fig. 3b shows that the datapoints are concentrated toward the lower left portion, thereby hinting that the lower surface coverage values could be better fitted by the model. This information can be utilized to rapidly screen between two dependent variables to see trends within the datapoints describing the electropolymerization process.

**Figure 4:**
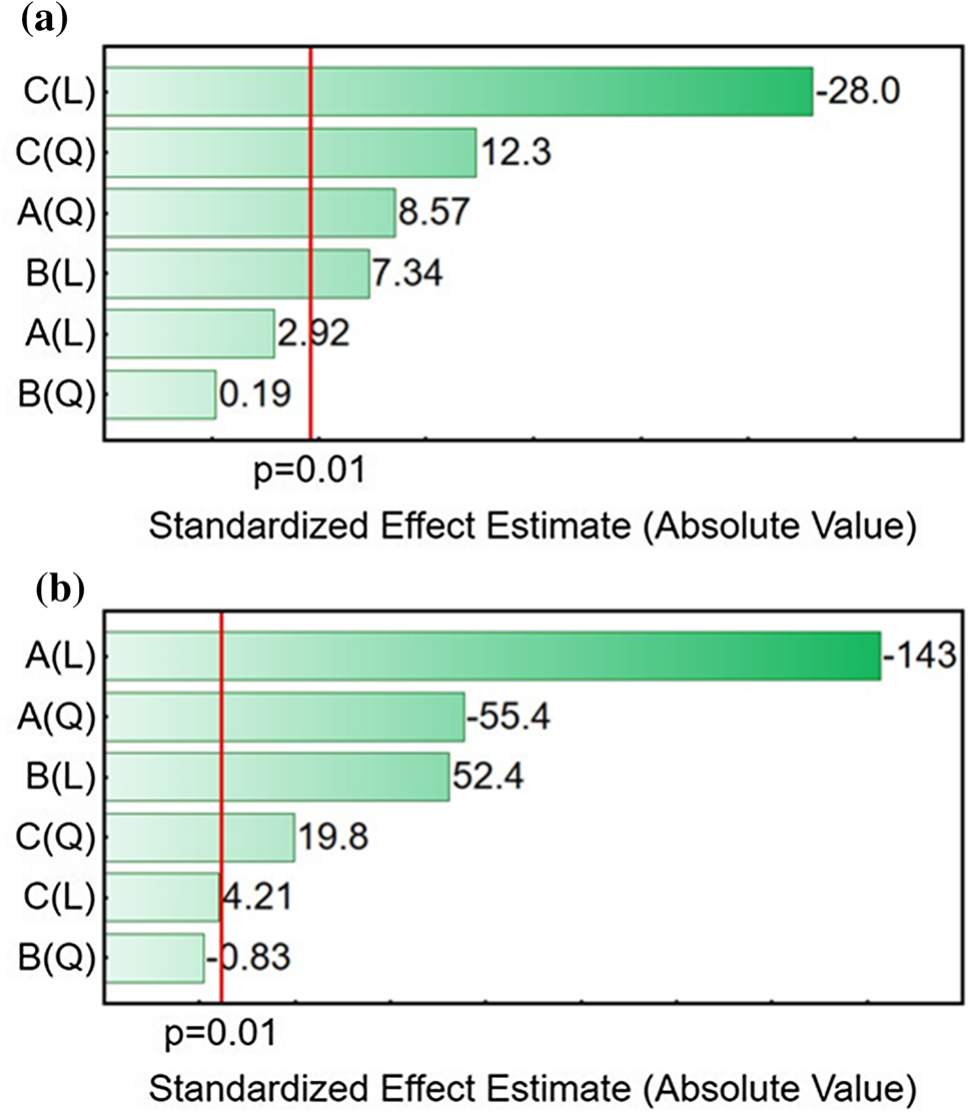
**a** Pareto Chart showing the statistical significance of each factor on the electroactive area,** b** Pareto chart showing the statistical significance of each factor on the surface coverage. A = scan rate; B = number of scans; C = monomer concentration

Pareto charts are used to display the effect of each factor within a statistical analysis. The chart shows the absolute values of the standardized effects ranking them in descending order. Typically, a standard *p*-value < 0.05 is used to discriminate between statistically significant and statistically insignificant factors. A preliminary evaluation of the electropolymerization process was carried out based on a *p* value < 0.05; at a later stage, the *p*-value was lowered to 0.01 in order to discriminate only the most statistically relevant results. Figure 4 reports the two Pareto charts resulting from the three-way ANOVA analysis. The vertical red line is used as a rapid visual tool to discriminate between statistically significant and not significant factors. Figure 4a reports the Pareto chart for the electroactive area (EA). A first glance at the graph already provides a clear hint that there is a large dependency of the electroactive area on the third factor (*C*), the concentration of the monomer. Moreover, it can be similarly assessed how the linear component is largely prevalent over the quadratic one, suggesting a potentially linear correlation between electroactive area and monomer concentration. Interestingly, a negative value of the standardized effect estimate suggests that an increase in the concentration of the monomer should lead to a decrease in the output of the dependent variable. This logically resonates with the fact that increasing concentrations of the active monomer tend to favor the formation of polymerized layers on the surface of the electrode, thus reducing the electroactive area.

This correlates well with the findings showcased by the Pareto chart, where the third factor, SP concentration, had the highest statistical significance on the dependent variable, i.e., the electroactive area, especially in its linear component.

Differently, the first factor, the scan rate, shows a predominant significance of its quadratic component. Referring back to the Randles–Sevcik equation, this is justified by the mathematical modeling that sees a quadratic correlation between the electroactive area and the scan rate, as $A \propto v^{\frac{1}{2}}$.

The second factor, the number of cycles, appears to be the least statistically significant on the output of our dependent variable. A weak linear proportionality can be inferred from the Pareto chart, while its quadratic component is the only factor that is deemed to be not statistically significant in this analysis.

Figure 4b reports the Pareto chart for the analysis of surface coverage. The graph visually confirms a dominant effect of factor *A*, the scan rate, on the output of the second variable, the surface coverage. Moreover, as it was the case in Fig. 4a, the standardized effect estimate is negative, suggesting that an increase in scan rate would negatively affect the surface coverage. Being the standardized effect reported in absolute values and being the scales that have been used for factors and output variables consistent between Fig. 4a and b, the same factors can be compared across the two graphs. For instance, Fig. 4b shows how the number of cycles (*B*) is much more relevant for the surface coverage, than it is for the electroactive area. We suggest that an increased number of scans effectively increase the thickness of the polymer film in the electrode surface, thus increasing the surface coverage. The electroactive area, however, is also dependent on the conformation of the film, be that ordered or disordered, and thus, it is less dependent on the number of scans but could be influenced by other factors.

### Response surface methodology

A complex system comprising multiple variables can be thoroughly studied by exploiting response surface methodology (RSM) that gives insights on the correlation between the independent factors and the resulting dependent variable. RSM is considered to be a surface placement method, where the topography of the chart determines the regions of optimal response [38]. The system can then be later optimized based on the critical values that are displayed as the output. RSM is a handy tool to reduce the number of experiments to be performed, thus saving resources and avoiding time-consuming operations [39].

Three-dimensional (3D) plots are obtained by applying RSM on the experimental design for each dependent variable. In this case, three 3D plots have been generated based on the combination of the three factors, namely scan rate, number of cycles and monomer concentration.

**Figure 5:**
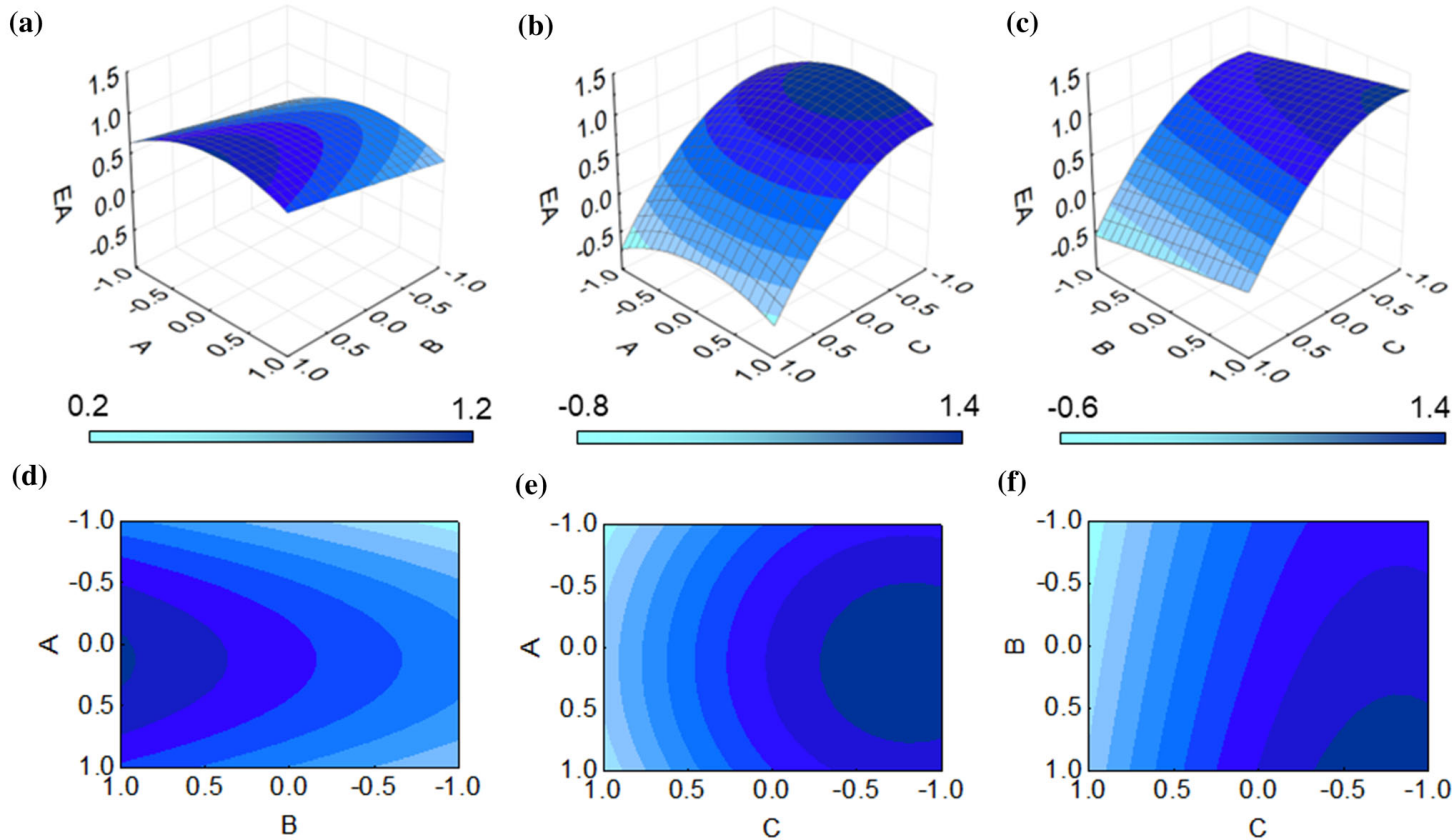
**a** 3D plots showing the combined effect of scan rate and number of scans on the electroactive area** b** 3D plots showing the combined effect of scan rate and monomer concentration on the electroactive area** c** 3D plots showing the combined effect of number of scans and monomer concentration on the electroactive area** d** Contour plot of electroactive area as a function of scan rate and number of scans **e** Contour plot of electroactive area as a function of scan rate and monomer concentration** f** Contour plot of electroactive area as a function of number of scans and monomer concentration

Figure 5 provides the RSM 3D plots for each combination of factors in the determination of the electroactive area and the relative 2D representation through contour plots.

Figure 5a and d shows the effect of scan rate and number of scans on the electroactive area. These two parameters were assessed as being less relevant than monomer concentration, as previously noticed in the Pareto chart. That can also be observed in the RSM plot that displays a quite flat profile, thus showing little variation in the output value on the z axis as a function of the two parameters on the x and y axis. The contour plot, however, allows us to identify specific areas where the combined contribution of the two parameters tends toward greater values of the z variable, thus minimizing the electroactive area. The 2D plot shows how higher number of scans tends to increase the output, while the best values of scan rate center around the intermediary value of 100 mv/s. This is also confirmed by the response surfaces of scan rate vs monomer concentration, shown in the 3D plot and the contour plot in Fig. 5b and e, respectively. Here the 3D plot reinforces the significant contribution of monomer concentration in determining the final electroactive area. The plot follows a much steeper curvature, also demonstrating a positive effect of the quadratic component of factor *C*, the monomer concentration. The contour plots show how the scan rate optimal points are centered around the intermediary value along the y axis, while the monomer concentration shifts the center point of the plot toward the right side, thus demonstrating how a lower concentration of the monomer is needed in order to maximize the output. The steep curvature in the RSM 3D plot is consistent in Fig. 5c where the electroactive area is evaluated as a function of number of scans and monomer concentration. Factor *C* clearly dominates the topography of this graph as well, and the resulting contour plot (Fig. 5f) is used to confirm the individual information on factor *A* and factor *C* that have been presented in the previous figures.

**Figure 6:**
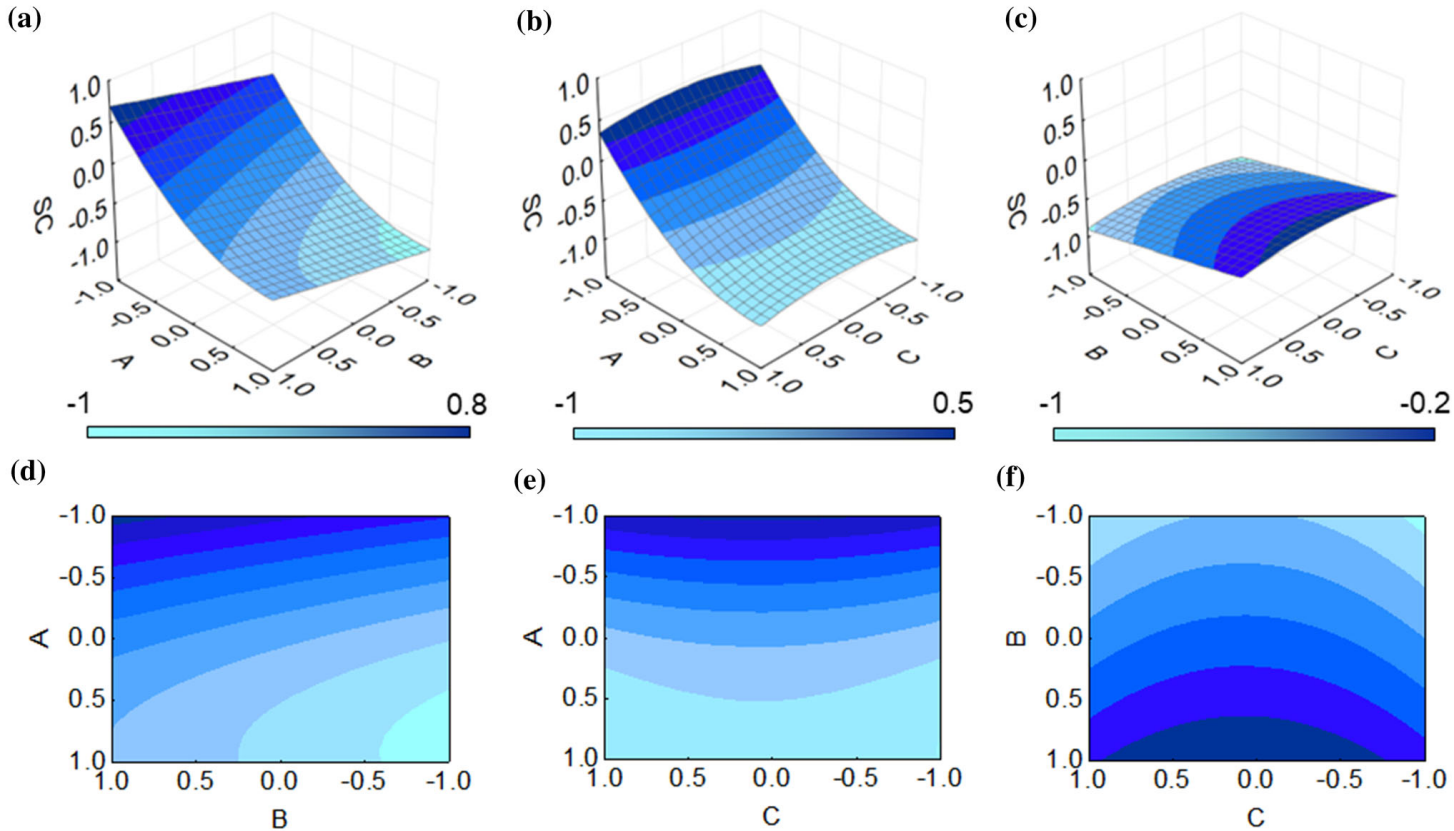
**a** 3D plots showing the combined effect of scan rate and number of scans on the surface coverage,** b** 3D plots showing the combined effect of scan rate and monomer concentration on the surface coverage,** c** 3D plots showing the combined effect of number of scans and monomer concentration on the surface coverage,** d** contour plot of surface coverage as a function of scan rate and number of scans, **e** contour plot of surface coverage as a function of scan rate and monomer concentration, **f** contour plot of surface coverage as a function of number of scans and monomer concentration

RSM was also applied to determine the effect of each factor on the resulting surface coverage. Electroactive area and surface coverage as output variables are reportedly correlated, but the information they can provide on the overall system is not redundant. In fact, surface coverage is known to factor in the thickness of the electrosynthesized polymer, thence providing hints on layers stacking. On the other hand, the electroactive area provides information regarding changes on the surface of the working electrode. In our model, it is shown that satisfactory results in minimizing the electroactive area are not directly followed by a maximization in surface coverage, as it could be expected for redundant variables. Figure 6 collects 3D RSM and contour plots for surface coverage, showing a marked difference with the results reported in Fig. 5 for the electroactive area. In this case, in fact, the plots show how the optimal conditions for maximizing surface coverage were far from the levels used when crafting the DoE. Figure 6a and d reports surface coverage as a function of scan rate and number of scans. The RSM 3D plot shows a noticeable negative influence of scan rate on the output, both in its linear and quadratic components. The number of scans also affected the resulting final surface coverage, although with a much more limited impact, showing a positive trend with the increase in the number of scans. The surface plot confirms the findings of the 3D plot showing a better outcome in the top left corner, where number of scans is at their maximum value and scan rate is at its minimum. Figure 6b and e collectively explores the influence of scan rate and monomer concentration on the final surface coverage. The effect of scan rate retains its importance with respect to the monomer concentration as well that has minimal impact on the 3D plot both in magnitude of *z*-variation and curvature of the graph. The contour plot confirms the almost negligible impact of factor *C*, showing the greatest *z* value at the top portion of the graph. Figure 6c and f displays the plot of the two least relevant factors in the analysis of surface coverage. These graphs have features that closely resemble that of Fig. 5a and d where the two least relevant factors for the minimization of electroactive area were shown. These plots confirm the findings obtained in the precedent figures. A higher number of cycles are thus expected to increase the surface coverage, while monomer concentration has even lesser impact, with satisfactory values obtained for the middle level 0 of monomer concentration. Analyzing response surfaces is important to bear in mind that these methods are not exempt from errors, as experimental data can’t always be fitted to second order polynomials [40]. However, contemporarily exploring two output variables allows to draw fast and simple considerations on the most relevant factors, thus potentially guiding future modifications of the DoE to have even more precise results.

## Conclusions

This study reports a detailed analysis of the electropolymerization of SP on the surface of glassy carbon electrodes. We demonstrated how QbD principles can help to guide the electropolymerization process by screening the most relevant variables to be controlled, thus potentially being of great help for researchers that are involved in electropolymerization processes. This technique is highly scalable and can be rapidly translated to other similar compounds or different electrode surfaces. The selection of the independent parameters to be analyzed is also dependent on the electropolymerization process, as other variables including pH and T of the solution could be included in the experimental matrix, if other parameters are deemed to be less important. Moreover, as different dependent variables can be chosen when evaluating the efficacy of the process, this experimental design can be tailored for specific uses (i.e., surface coverage is of great importance whether the synthesized polymer should be employed as a coating, thus requiring uniformity). Finally, this study allowed us to derive theoretical values that could lead to an optimization of the electropolymerization process according to the two key parameters that have been chosen as dependent variables, namely electroactive area and surface coverage. However, since these values fall outside of the range that was chosen for the experimental matrix for both independent variables, we could only suggest that a significantly higher number of scans could lead to a better electropolymerization process. These are, however, theoretical critical values that will warrant further investigation; thus, a full optimization and its validation would be thoroughly explored in future outreaches.

The use of quality-by-design approaches in studying electropolymerization processes is not often described in the literature. To the best of our knowledge, this is the first report of an application of systematic design of experiments and multifactorial analysis in the study of polyscopoletin electrosynthesis. This exploratory study is intended to stimulate further research in the direction of rational design of experimental protocols of electropolymerization processes, thereby aiming to save time and resources.

## Supplementary Information

Below is the link to the electronic supplementary material.

Supplementary file1 (DOCX 17 KB)

## Data Availability

The data presented in this study are openly available online on OSF at osf.io/5en8a.
