# Supplementary material for: An insight into polyscopoletin electrosynthesis by a quality-by-design approach
Source: J Mater Sci. 2022 Jun 22;57(25):12161–75. doi: 10.1007/s10853-022-07349-8 (PMC9215150; doi:10.1007/s10853-022-07349-8)
Supplement: Supplementary file 1 — Supplementary file1 (DOCX 17 KB) [file 10853_2022_7349_MOESM1_ESM.docx]

**Supplementary Information**

**Table S1.** ANOVA Table for Factor Components (EA)

| Factors | SS | df | MS | F | p-value |
| --- | --- | --- | --- | --- | --- |
| A (L) | 0.046396 | 1 | 0.046396 | 8.5157 | 0.043323 |
| A (Q) | 0.400585 | 1 | 0.400585 | 73.5252 | 0.001016 |
| B (L) | 0.293199 | 1 | 0.293199 | 53.8150 | 0.001838 |
| B (Q) | 0.000202 | 1 | 0.000202 | 0.0371 | 0.856706 |
| C (L) | 4.271556 | 1 | 4.271556 | 784.0208 | 0.000010 |
| C (Q) | 0.830585 | 1 | 0.830585 | 152.4494 | 0.000247 |
| Lack of Fit | 0.272620 | 6 | 0.045437 | 8.3397 | 0.029721 |
| Pure Error | 0.021793 | 4 | 0.005448 |  |  |
| Total SS | 6.211101 | 16 |  |  |  |

**Table S2.** ANOVA Table for Factor Components (SC)

| **Factors** | **SS** | **df** | **MS** | **F** | **p-value** |
| --- | --- | --- | --- | --- | --- |
| A (L) | 3.431024 | 1 | 3.431024 | 20370.58 | 0.000000 |
| A (Q) | 0.517260 | 1 | 0.517260 | 3071.06 | 0.000001 |
| B (L) | 0.462780 | 1 | 0.462780 | 2747.60 | 0.000001 |
| B (Q) | 0.000115 | 1 | 0.000115 | 0.68 | 0.454962 |
| C (L) | 0.002981 | 1 | 0.002981 | 17.70 | 0.013619 |
| C (Q) | 0.065764 | 1 | 0.065764 | 390.45 | 0.000039 |
| Lack of Fit | 0.482476 | 6 | 0.080413 | 477.42 | 0.000012 |
| Pure Error | 0.000674 | 4 | 0.000168 |  |  |
| Total SS | 4.946444 | 16 |  |  |  |
